# HbA1C Variability Is Strongly Associated With the Severity of Cardiovascular Autonomic Neuropathy in Patients With Type 2 Diabetes After Longer Diabetes Duration

**DOI:** 10.3389/fnins.2019.00458

**Published:** 2019-05-14

**Authors:** Yun-Ru Lai, Chih-Cheng Huang, Wen-Chan Chiu, Rue-Tsuan Liu, Nai-Wen Tsai, Hung-Chen Wang, Wei-Che Lin, Ben-Chung Cheng, Yu-Jih Su, Chih-Min Su, Sheng-Yuan Hsiao, Pei-Wen Wang, Jung-Fu Chen, Cheng-Hsien Lu

**Affiliations:** ^1^Department of Biological Science, National Sun Yat-sen University, Kaohsiung, Taiwan; ^2^Department of Neurology, Kaohsiung Chang Gung Memorial Hospital, Chang Gung University College of Medicine, Kaohsiung, Taiwan; ^3^Department of Internal Medicine, Kaohsiung Chang Gung Memorial Hospital, Chang Gung University College of Medicine, Kaohsiung, Taiwan; ^4^Department of Neurosurgery, Kaohsiung Chang Gung Memorial Hospital, Chang Gung University College of Medicine, Kaohsiung, Taiwan; ^5^Department of Radiology, Kaohsiung Chang Gung Memorial Hospital, Chang Gung University College of Medicine, Kaohsiung, Taiwan; ^6^Emergency Medicine, Kaohsiung Chang Gung Memorial Hospital, Chang Gung University College of Medicine, Kaohsiung, Taiwan; ^7^Center for Shockwave Medicine and Tissue Engineering, Kaohsiung Chang Gung Memorial Hospital, Chang Gung University College of Medicine, Kaohsiung, Taiwan; ^8^Department of Neurology, Xiamen Chang Gung Memorial Hospital, Xiamen, China

**Keywords:** cardiovascular autonomic neuropathy, composite autonomic scoring scale, HbA1c variability, long diabetes duration, type 2 diabetes

## Abstract

**Background:**

Variability in the glycated hemoglobin (HbA1c) level is associated with a higher risk of microvascular complications in patients with type 2 diabetes. We tested the hypothesis that HbA1c variability is not only strongly associated with the presence but also the degree of severity of cardiovascular autonomic neuropathy (CAN) in patients with long diabetes durations (more than 10 years).

**Methods:**

For each patient, the intrapersonal mean, standard deviation (SD), and coefficient of variation (CV) for HbA1c were calculated using all measurements obtained 3 years before the study. We constructed the composite autonomic scoring scale (CASS) as a measure of the severity of cardiovascular autonomic functions. Stepwise logistic regression and linear regression analyses were performed to evaluate the presence of CAN and the influence of independent variables on the mean CASS, respectively.

**Results:**

Those with CAN had a higher mean age, a higher low-density lipoprotein cholesterol (LDL-C), HbA1c-SD, HbA1c-CV, mean HbA1c, and index HbA1c, higher prevalence of retinopathy as the underlying disease, and lower high-density lipoprotein (HDL) levels. Stepwise logistic regression showed that HbA1c-SD and retinopathy were risk factors that were independently associated with the presence of CAN. Mean HbA1c, HbA1c-CV, HbA1c-SD, and index HbA1c were positively correlated with mean CASS, and a multiple linear regression analysis revealed that HbA1c-SD was independently associated with the mean CASS.

**Conclusion:**

HbA1c variability is strongly associated with not only the presence but also the degree of severity of CAN. A longitudinal study is required to confirm whether controlling blood glucose level is effective in reducing CAN progression.

## Background

Diabetic cardiovascular autonomic neuropathy (CAN) is common but is one of the most overlooked complications of diabetes (Ewing et al., 1980). Autonomic nervous systems, including parasympathetic and sympathetic nervous systems, innervate various organ systems and modulate their function. CAN indicates a length-dependent pattern of the disease (Papanas and Ziegler, 2015). The vagus nerve, responsible for approximately 75% of the parasympathetic activity in humans, can be damaged in the early phase of CAN (Ewing et al., 1985), resulting in decreased parasympathetic activity and contributing to sympathetic predominance. As the disease progresses, sympathetic denervation occurs in the late stage of CAN. Therefore, it has a wide spectrum of clinical presentation.

There is a wide variation in the prevalence of CAN, depending on the definition and criteria used for diagnosis. Estimating the severity of CAN is also a formidable challenge for clinicians. Although there are no uniform criteria for diagnosing or staging CAN (Tesfaye et al., 2010; Rolim et al., 2013), the recent Toronto Consensus recommends the use of four cardiovascular reflex tests [deep breathing, Valsalva maneuver (VM), orthostatic (30:15) and orthostatic hypotension] and frequency-domain tests as the most sensitive and specific method of assessing the presence of CAN in patients with diabetes (Spallone et al., 2011). Further, Phillip Low constructed the composite autonomic scoring scales (CASS) for laboratory quantification of generalized autonomic failure (Low, 1993) and the CASS have been proven useful for estimating the severity of CAN (Huang et al., 2016).

There are several well-known risk factors associated with CAN development, including age, diabetes duration, glycemic control, hypertension, and dyslipidemia, body mass index (BMI), and development of other microvascular complications (e.g., retinopathy, proteinuria) (Dafaalla et al., 2016). Glycemic variability (GV) is a term used to describe the impairment of glycemic control. Multiple measures of GV have been suggested as potential predictors for CAN, and long-term GV assessment using HbA1c variability is significantly associated with the presence of CAN (Fleischer, 2012; Jun et al., 2015; Yang et al., 2018).

Cardiovascular autonomic neuropathy has a strong influence on various cardiovascular diseases and leads to severe morbidity and mortality in patients with diabetes (Vinik et al., 2003; Vinik and Ziegler, 2007). To date, there is paucity of information on the estimation of the overall severity of CAN using the CASS and its relationship between chronic glycemic impairment and different cardio-metabolic parameters in patients with long-duration type 2 diabetes. Scientists have been exploring the role of chronic GV in the subsequent severity of CAN and developing therapeutic strategies that may be beneficial in patients with diabetes. Therefore, potential risk factors need to be delineated to determine patients who are most appropriate for aggressive treatment. In this study, we tested the hypothesis that chronic GV is not only a risk factor for the presence of CAN but is also associated with the severity of CAN in patients with type 2 diabetes. The successful translation of these approaches offers the promise of reducing microvascular complications and improving the quality of life of patients with type 2 diabetes.

## Patients and Methods

### Patients

**FIGURE 1:**
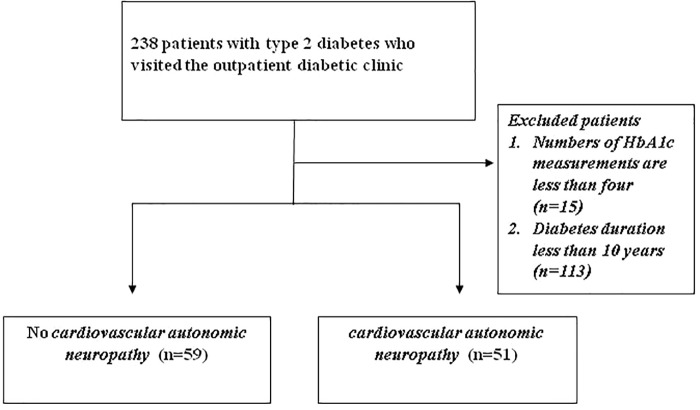
Enrollment of patients.

A total of 238 patients (≥20 years old) with type 2 diabetes who visited the outpatient diabetic clinic at Kaohsiung Chang Gung Memorial Hospital in Taiwan were identified from our previous cross-sectional prospective study (IRB 96-1361B, 99-2276C) (Huang et al., 2016). The exclusion criteria included (1) number of HbA1c measurements less than four, (2) diabetes duration less than 10 years; (3) moderate-to-severe heart failure [New York Heart Association (NYHA) class III and IV]; and (4) any type of arrhythmia that prevents the analysis of heart rate variability, or pacemaker implantation due to any cause. Thus, only 110 participants were enrolled in the study (Figure 1). The study was approved by the Ethics Committee of Chang Gung Memorial Hospital Institutional Review Board (201701243B0 and 201800388B0C501).

### Baseline Clinical and Laboratory Measurements

All patients underwent complete neurological and physical examinations upon enrollment and at their subsequent follow-ups at the outpatient clinic. A detailed medical history regarding prior use of medications was obtained from the patients and their families through standardized questions. Demographic data, including age, sex, duration of diabetes (years), BMI, systolic, and diastolic blood pressure (BP) during autonomic function testing, underlying diseases [hypertension, coronary artery disease (CAD), ischemic stroke, and diabetic retinopathy (DR)], and laboratory parameters were obtained at baseline.

### Assessment of Glycemic Variability

For each patient, the intrapersonal mean, SD, and coefficient of variation [CV = HbA1c-SD/(0.1 × mean HbA1c)] of HbA1c was calculated using all measurements obtained 3 years before the study. The HbA1c-SD was considered a measure of GV and the CV, a normalized measure of GV. Because the number of individual visits could influence the HbA1c-SD (with fewer visits likely to artificially inflate SD), we defined the adjusted SD of HbA1c as the SD of HbA1c divided by [n/(n-1)]^0.5^ (where n is the number of HbA1c measurements), to minimize any effect of different values of HbA1c on the ones calculated (Kilpatrick et al., 2008). Our study was repeated using adjusted HbA1c-SD instead of HbA1c-SD.

### Assessment and Scoring of Cardiovascular Autonomic Functions

All subjects underwent a standardized evaluation of the cardiovascular autonomic function, as described by Low (2003). The test battery consisted of the heart rate response to deep breathing (HR_DB), Valsalva ratio (VR), and baroreflex sensitivity (BRS). The heart rate was derived using a continuously recorded standard three-lead electrocardiography (Model 3000; Ivy Biomedical, Branford, CT, United States). Arterial BP was continuously measured at the finger, using beat-to-beat photoplethysmographic recordings (Finameter Pro; Ohmeda, Englewood, OH, United States). The tests were done between 9:00 am to noon for all patients. No coffee, food, alcohol, or nicotine was permitted 4 h before the tests. Patients on medications known to cause orthostatic hypotension or otherwise affecting autonomic testing were asked to stop taking the drug for five half-lives, provided that it was not harmful to the patient’s well-being. For patients on β-blockers for BP control, the drug was omitted on the day of the study and resumed after the test. The detailed computing of HR_DB and VR were as described by Low (2003). To quantify BRS, a linear regression analysis was performed between systolic BP and RR interval (RRI) changes during the early phase II of VM. In this phase, there was a progressive decrease in systolic BP due to reduced preload (venous return) and stroke volume with associated tachycardia (gradual shortening of RRI).

To improve the assessment of laboratory grading of CAN, we constructed a CASS for laboratory quantification of CAN according to our previous diabetic study (Huang et al., 2016). The severity of CAN was assessed using the cardiovagal and adrenergic sub-scores of the CASS. In this study, the scale was modified in the adrenergic sub-score since the 5-min head-up tilt test (HUTT) was not done in the current study. Thus, the CASS version used here allotted 3 points instead of 4 for the adrenergic domain. Further, patients with a CASS score ≥2 were defined as having CAN (Huang et al., 2016).

### Statistical Analysis

Data are expressed as means ± standard derivations (SDs) or medians (interquartile ranges). Categorical variables were compared using Chi-square or Fisher’s exact tests. Continuous variables that were not normally distributed in the Kolmogorov–Smirnov test were logarithmically transformed to improve normality and compared. Four separate statistical analyses were performed. Firstly, patients were stratified into two groups according to the presence or absence of CAN. One-way analysis of covariance (ANCOVA) was used to compare parameters of cardiovascular autonomic study with age as the added covariate. Secondly, the risk factors for the presence of CAN including sex and baseline characteristics, underlying diseases, and parameters of laboratory testing were analyzed using stepwise logistic regression. Thirdly, correlation analysis was used to evaluate the relationship between the CASS and variables that included age, diabetes duration, BMI, waist circumference, systolic and diastolic BP, and peripheral blood studies for vascular risks. Fourthly, stepwise models of multiple linear regression analysis were used to evaluate the influence of independent variables on the mean CASS. All statistical analyses were conducted using the SAS software package, version 9.1 (2002, SAS Statistical Institute, Cary, North Carolina).

## Results

### General Characteristics of the Patients With Diabetes

The 110 patients with diabetes included 45 women and 65 men. Patient characteristics, baseline underlying diseases and laboratory data at assessment are presented in Table 1, stratified into two groups according to the presence or absence of CAN. Those with CAN had higher mean age (*P* = 0.002), higher low-density lipoprotein cholesterol (LDL-C), HbA1c-SD, HbA1c-CV, mean HbA1c and index HbA1c (*P* = 0.04, *P* = 0.003, *P* = 0.009, *P* = 0.009, and *P* = 0.01, respectively), higher prevalence of retinopathy as the underlying disease (*P* < 0.0001) and lower high-density lipoprotein (HDL) levels (*P* = 0.02). Significant variables used in the stepwise logistic regression model included mean age, baseline LDL-C, HbA1c-SD, CV HbA1c, mean HbA1c, index HbA1c, and HDL level and the presence of retinopathy as the underlying disease. After analysis of all the aforementioned variables, only HbA1c-SD (*P* = 0.007, OR = 10.1, 95% CI = 1.90–54.4) and presence of retinopathy (*P* < 0.0001, OR = 731.1, 95% CI = 67.9–7869.3) were independently associated with the presence of CAN.

**Table 1:** Baseline characteristics of patients with Type 2 diabetes.

	No CAN (*n* = 59)	CAN (*n* = 51)	Crude *P*-value	Adjusted OR (95% CI)	Adjusted *P*-value
Characteristics					
Age (year)	62.2 ± 8.8	67.2 ± 7.6	0.002**		
Sex (male/female)	34/25	31/20	0.74		
Diabetes duration (year)	17.4 ± 5.8	17.4 ± 6.3	1.0		
Body mass index	25.6 ± 3.3	26.2 ± 3.7	0.35		
SBP (mmHg)	140.5 ± 19.7	144.4 ± 19.1	0.29		
DBP (mmHg)	74.5 ± 11.4	75.5 ± 11.2	0.65		
Baseline underlying disease					
Retinopathy, n (%)	2 (3%)	43 (84%)	<0.0001***	731.1 (67.9–7869.3)	<0.0001***
Proteinuria, n (%)	38 (64%)	37 (73%)	0.21		
Coronary heart disease (%)	10 (17%)	11 (22%)	0.54		
Ischemic stroke (%)	13 (22%)	12 (24%)	0.85		
Laboratory test findings					
Total cholesterol (mmol/L)	152.9 ± 27.8	157.1 ± 27.4	0.43		
Triglyceride (mmol/L)	133.1 ± 75.4	142.0 ± 73.9	0.54		
HDL-C (mmol/L)	55.7 ± 14.9	49.2 ± 13.0	0.02*		
LDL-C (mmol L)	70.9 ± 24.8	80.7 ± 25.4	0.04*		
Index HbA1c (%) 6.9 ± 0.9 7.4 ± 0.9 7.3 ± 1.1 7.4 ± 1.3 0.171	7.0 ± 1.0	7.5 ± 1.0	0.01*		
Mean HbA1c (%)	7.4 ± 1.0	7.9 ± 1.1	0.009**		
HbA1c-SD (%)	0.6 ± 0.4	0.9 ± 0.5	0.003**	10.1 (1.90–54.4)	0.007**
HbA1c-CV	9.0 ± 4.8	12.1 ± 7.4	0.009**		
Type of diabetes treatment					
OHA only	37	28	0.71		
Insulin only	14	26			
No treatment	2	4			
Other concomitant medications					
ACE inhibitor or ARB	40	41	0.57		
Beta-blocker	16	16	0.76		
Calcium channel blocker	21	18	0.31		
Diuretics	27	23	0.23		
Lipid-lowering medications	40	40	0.45		


*Data are presented as means ± standard deviations or n (%). ^∗^*P* < 0.05; ^∗∗^*P* < 0.01; ^∗∗∗^*P* < 0.001. CAN, cardiac autonomic neuropathy; n, number of cases; SBP, systolic blood pressure; DBP, diastolic blood pressure; HDL-C, high-density lipoprotein cholesterol; LDL-C, low-density lipoprotein cholesterol; HbA1c, glycohemoglobin; CV, coefficient of variation; SD: standard deviations; OHA, oral hypoglycemic agent; ACE, angiotensin-converting enzyme; ARB, angiotensin II receptor blocker.*

### Parameters of Cardiovascular Autonomic Study Between Patients With or Without CAN

Cardiovascular autonomic study in patients with type 2 diabetes stratified into two groups according to the presence or absence of CAN is presented in Table 2. The parameters of cardiovascular autonomic study were compared by ANCOVA after controlling for age. Those with CAN had higher CASS (*P* < 0.0001) and lower HR_DB (*P* < 0.0001), VR *P* < 0.0001, and BRS *P* < 0.0001.

**Table 2:** Baseline cardiovascular autonomic study with Type 2 diabetes.

	No CAN (*n* = 59)	CAN (*n* = 51)	*F*^†^	*P*-value^†^
Composite Autonomic Scoring Scale	0.7 ± 0.4	2.9 ± 1.3	119.3	<0.0001***
HR_DB (beats/min)	8.3 ± 4.7	4.3 ± 2.6	46.2	<0.0001***
Valsalva ratio	1.3 ± 0.1	1.2 ± 0.1	22.5	<0.0001***
Baroreflex sensitivity	1.6 ± 0.9	0.9 ± 0.8	13.9	<0.0001***


*Data are presented as means ± standard deviations or n (%). ^∗^*P* < 0.05; ^∗∗^*P* < 0.01; ^∗∗∗^*P* < 0.001. Parameters of cardiovascular autonomic study were compared by ANCOVA after controlling for age. *F*^†^ and *P*^†^ represent the comparison between CAN vs.no CAN group. n, number of cases; HR_DB, heart rate response to deep breathing; CAN, cardiac autonomic neuropathy.*

### Effect of Glycemic Variability and Other Vascular Risk Factors on Composite Autonomic Scoring

Correlation analysis parameters used to test the influence of GV and other vascular risk factors on CASS are listed in Table 3. The significant statistical results (correlation coefficient, *P*-value) were as follows: mean HbA1c (%) (*r* = 0.220, *P* = 0.028), HbA1c-CV (*r* = 0.197, *P* = 0.05), HbA1c-SD (*r* = 0.232, *P* = 0.02), index HbA1c (%) (*r* = 0.207, *P* = 0.039). All the correlation coefficients in mean HbA1c (%), HbA1c-CV, HbA1c-SD, and index HbA1c (%) indicate a weak positive linear relationship (*r* < 0.4).

**Table 3:** Correlation analysis of composite autonomic scoring scale in patients with type 2 diabetes.

Variables	Composite Autonomic Scoring Scale (*n* = 110)	
	*r*	*P*-value
Age (year)	–0.123	0.089
Diabetes duration (year)	0.036	0.725
Body mass index	0.042	0.675
SBP (mmHg)	0.121	0.230
DBP (mmHg)	0.021	0.839
Total cholesterol (mmol/L)	0.149	0.140
Triglyceride (mmol/L)	0.024	0.816
HDL-C (mmol/L)	–0.101	0.315
LDL-C (mmol/L)	0.194	0.053
Index HbA1c (%)	0.207	0.039*
Mean HbA1c (%)	0.220	0.028*
HbA1c-CV	0.197	0.05*
HbA1c-SD	0.232	0.02*


*^∗^P < 0.05. *r*, correlation coefficient. n, number of cases; SBP, systolic blood pressure; DBP, diastolic blood pressure; HDL-C, high-density lipoprotein cholesterol; LDL-C, low-density lipoprotein cholesterol; HbA1c, glycohemoglobin; CV, coefficient of variation.*

### Clinical Factors Are Significantly Associated With Composite Autonomic Scoring

Effects of the variables on the CASS in patients with type 2 diabetes according to correlation analysis are listed in Table 4. Statistical analysis was subsequently carried out to decipher the relationship between the augmented CASS in patients with diabetes and GV. Based on correlation analysis, our results revealed that mean HbA1c, HbA1c-SD, and index HbA1c were significantly correlated with CASS (Table 3). We further employed multiple linear regression analysis on the aforementioned variables to identify the crucial determinant that underlies the augmented CASS in patients with diabetes. Results from the model analysis (Table 4) revealed that only HbA1c-SD were significantly associated with the CASS.

**Table 4:** Effects of the variables on composite autonomic scoring scale in patients with type 2 diabetes according to correlation analysis.

	Regression coefficient	Standard error	*P*-value
Constant	1.148	0.299	<0.0001
HbA1c-SD	0.784	0.327	0.018*
Mean HbA1c	0.07	0.222	0.753
Index HbA1c	0.209	0.222	0.348


*Regression coefficient for each individual variable. ^∗^*P* < 0.05. HbA1c, glycohemoglobin; CV, coefficient of variation; SD, standard deviations.*

## Discussion

### Major Findings of Our Study

To our knowledge, this is the first study to confirm the hypothesis that HbA1c variability is not only strongly associated with the presence but also the degree of severity of CAN, in patients with long-duration type 2 diabetes. We examined the role of HbA1c variability on the presence and severity of CAN to obtain three major findings.

First, those with CAN had higher mean age, higher LDL-C, HbA1c-SD, HbA1c-CV, mean HbA1c, and index HbA1c, higher prevalence of retinopathy as the underlying disease, and lower HDL levels. Second, HbA1c-SD and the presence of retinopathy were independently associated with the presence of CAN. Finally, although mean HbA1c (%), HbA1c-CV, HbA1c-SD, and index HbA1c were positively correlated with the CASS score in correlation analysis, the correlation coefficient indicates a weak positive linear relationship (*r* < 0.4). Multiple linear regression analysis revealed that HbA1c-SD was independently associated with the mean CASS.

### The Role of Persistent Poor Glucose Control and Glycemic Variability in Diabetic Complications

Aggressive control of blood glucose is pivotal for patients with type 2 diabetes, and can prevent microvascular and macrovascular complications (Brownlee and Hirsch, 2006; Siegelaar et al., 2010). Short-term GV indicated that patients with similar mean glucose or HbA1c values can show markedly different daily glucose profiles (Brownlee and Hirsch, 2006; Siegelaar et al., 2010). Either fluctuating or persisting high glucose levels can induce oxidative stress, contribute to endothelial dysfunction, and finally result in diabetic complications (Brownlee and Hirsch, 2006; Siegelaar et al., 2010). Recently, clinical evidence also demonstrated that long term GV might be related to microvascular complications in type 2 diabetes (Fleischer, 2012; Jun et al., 2015; Wei et al., 2016; Yang et al., 2018).

### Glycemic Variability and Other Potential Risk Factors Associated With the Development of CAN

The pathophysiological mechanism of CAN development is multifactorial, and several studies reported the important role of cardiovascular risk factors, such as systolic BP, triglyceride levels, BMI, and smoking, in the development of CAN (Rolim et al., 2008; Dafaalla et al., 2016). Even more important, however, were the results of one clinical study that concluded that intensified multifactorial intervention (hyperglycemia, hypertension, dyslipidemia, and microalbuminuria) in patients with type 2 diabetes reduced the risk of CAN progression by 68% (Gæde et al., 1999). Another study also enhanced the role of intensive control in preventing and slowing the progression of CAN in patients with type 1 diabetes (The Diabetes Control and Complications Trial Research Group, 1998).

### Glycemic Variability and the Severity of CAN

The risk factors for CAN that were identified may provide important clues to etiologies, or merely reflect chance associations. Only when the same risk factor is found to be associated with the severity of CAN consistently, can it be concluded that there is a plausible mechanistic link between the risk factor and the disease progression or prevention. To the best of our knowledge, only limited studies have investigated the impact of GV on the severity of CAN in type 2 diabetes (Jun et al., 2015; Yang et al., 2018). One study of 110 patients with type 2 diabetes who underwent both short term and long term GV (HbA1c variability) demonstrated that only long term GV (HbA1c variability) was significantly associated with the presence of CAN by logistic regression analysis, and higher long term GV (HbA1c variability) had an increased risk of advanced CAN (Jun et al., 2015). In this study, the authors only investigate the role of both short term and long term GV without other vascular risk factors in the severity of CAN which was graded using ordinal logistic regression analysis. Another study enrolled 681 subjects (294 normal, 318 early, and 69 severe CAN) and the severity of CAN was categorized as normal, early, or severe according to the cardiac autonomic reflex tests (CARTs) score. The study was repeated using HbA1c-CV or adj-HbA1c-SD instead of HbA1c-SD as a measure of HbA1c variability and performed to test the association between the CAN and HbA1c variability indices by multivariable logistic regression analysis (Yang et al., 2018). In comparison to the patients described in the previous two studies, our patients were older and had longer diabetes durations and HbA1c variability was not only strongly associated with the presence but also the degree of severity of CAN. The discrepancy between these studies and our study may be attributed to different enrollment criteria (e.g., age and diabetes duration of patients, the time period before the study for measurement of HbA1c variability was obtained, the minimum number of HbA1c measurements), diagnostic criteria for CAN, and statistical methods used for assessing the severity of CAN (ordinal logistic regression, multiple logistic regression and linear regression).

### Study Limitations

This study has several limitations. Firstly, although the measurement of HbA1c variability was obtained 3 years before the study and there was a relationship between HbA1c variability and the degree of severity of CAN in patients with diabetes in this observational study, our study does not allow inferring causality or whether controlling blood glucose can improve CAN, even though it can be a starting point for future studies. A prospective longitudinal study is necessary to evaluate the efficiency of CASS as a scale of severity in clinical follow-up. Secondly, we excluded patients whose disease duration for diabetes was less than 10 years and the number of HbA1c measurements were less than four. Thus, there is uncertainty in assessing the role of chronic glycemic impairment and other cardio-metabolic parameters in the patients with type 2 diabetes who were omitted. Thirdly, the severity of CAN was assessed using the modified CASS rather than the original version because the 5-min HUTT was not done in this study. We aimed to set up a highly effective autonomic screening service at the out-patient clinic. The tests of deep breathing and VM can be conducted only by one technician within 10–15 min. One may argue about the validity of the modified CASS. Table 5 lists the comparison between the modified and the original versions of CASS. The only difference is in the three points versus four points in the adrenergic sub-score. If the 5-min HUTT is done, the adrenergic sub-score may reach to four points in a patient with extremely severe autonomic failure. In other words, the limitation of modified CASS is mainly in its resolution in differentiating severe from extremely severe patients. However, it is uncommon for patients with diabetes to have severe autonomic failure. It was reported as occurring in about 1–5% of the diabetic population (Low, 1996). In the current study, only four patients had severe adrenergic failure (three points in adrenergic sub-score). The four patients are those who may have different scores between the two versions of CASS. In other words, it is certain that the other 106 patients (96%) would have the same scores even if the original version of CASS was used. Therefore, we suppose that the impact of alteration in CASS is trivial to this study. The final limitation is that only cardiovagal and adrenergic domains were assessed, and the sympathetic sudomotor function was not included in this study. Thus, all the variability in severity found in CAN may not have been accounted for and this may produce statistical bias. Other quantitative approaches to assess the sudomotor function, such as quantitative sudomotor axon reflex test or sudoscan, should be taken into account for future studies.

**Table 5:** Comparison of composite autonomic scoring Scale (CASS) and Modified CASS (sub-scores in cardiovagal and adrenergic domains).

CASS		Modified CASS	
**Cardio-vagal**		**Cardio-vagal**	

0	Normal	0	The same with CASS
1	HR_DB mildly reduced but >50% of minimum	1	
2	HR_DB reduced to <50% of minimum or HR_DB + VR reduced	2	
3	Both HR_DB and VR reduced to <50% of minimum	3	

**Adrenergic**		**Adrenergic**	

0	Normal	0	Normal
1	Early phase II reduction > 20 but <40 mmHg MBP (30–40 if >50 years).	1	Early phase II reduction > 20 but <40 mmHg MBP (30–40 if >50 years)
	Late phase II does not return to baseline		Late phase II does not return to baseline.
	Pulse pressure reduction to ≤50% of baseline.		Pulse pressure reduction to ≤50% of baseline.
2	Early phase II reduction >40 mmHg MBP.	2	Early phase II reduction >40 mmHg MBP.
3	Early phase II reduction >40 mmHg + absent late phase II and phase IV.	3	Early phase II reduction >40 mmHg + absent late phase II and phase IV.
4	Criteria for 3 + orthostatic hypotension (SBP decrease ≥30 mmHg or MBP ≥ 20 mmHg).		


*Modified CASS allots 3 points instead of 4 for the adrenergic domain. HR_DB, heart rate response to deep breathing; VR, Valsalva ratio; SBP, systolic blood pressure; MBP, mean blood pressure.*

## Conclusion

Based on our results, HbA1c variability is not only strongly associated with the presence but also the degree of severity of CAN. As HbA1c variability is considered a prognostic factor, a longitudinal study is required in order to state that controlling blood glucose is effective in reduce CAN progression.

## Data Availability

The data from this study can be acquired from the corresponding author upon reasonable request.

## Ethics Statement

This study conformed to the guidelines of the Declaration of Helsinki, and the study has been approved by the Institutional Review Board of Chang Gung Memorial Hospital (201701243B0 and 201800388B0C501).

## Author Contributions

Y-RL participated in the design of the study and drafted the manuscript. W-CC, C-CH, R-TL, N-WT, H-CW, W-CL, B-CC, Y-JS, C-MS, S-YH, P-WW, and J-FC participated in the sequence alignment and clinical evaluation of patients. C-CH performed the statistical analysis. C-HL conceived the idea for the study, and participated in its design and coordination, and helped to draft the manuscript. All authors read and approved the final manuscript.

## Conflict of Interest Statement

The authors declare that the research was conducted in the absence of any commercial or financial relationships that could be construed as a potential conflict of interest.

## Abbreviations

BMI: body mass index
BRS: Baroreflex sensitivity
CAN: cardiac autonomic neuropathy
DBP: diastolic blood pressure
HbA1c: glycohemoglobin
HDL-C: high-density lipoprotein cholesterol
HR_DB: heart rate response to deep breathing
LDL-C: low-density lipoprotein cholesterol
SBP: systolic blood pressure
SD: standard deviation
VR: Valsalva ratio
